# Spatial disruptions and the embodied Self in schizophrenia: toward a developmental framework

**DOI:** 10.1038/s41537-026-00749-8

**Published:** 2026-05-05

**Authors:** Andrea Raballo, Antonio Preti, Michele Poletti

**Affiliations:** 1 Chair of Psychiatry, Faculty of Biomedical Sciences, University of Southern Switzerland, Lugano, Switzerland; 2 Cantonal Sociopsychiatric Organisation, Mendrisio, Switzerland; 3 Department of Neuroscience, University of Turin, Turin, Italy; 4 Department of Mental Health and Pathological Addiction, Child and Adolescent Neuropsychiatry Service, Azienda USL-IRCCS di Reggio Emilia, Reggio Emilia, Italy

**Keywords:** Schizophrenia, Human behaviour

## Abstract

Converging evidence indicates that schizophrenia reshapes the embodied structure of subjectivity, profoundly altering how individuals experience their bodies and surrounding space. This Perspective proposes a neurodevelopmental framework linking measurable distortions of personal space (PS) and peripersonal space (PPS) to deeper phenomenological disruptions of lived spatiality. Experimental findings consistently show an enlarged PS and a contracted PPS, maybe reflecting an excessive feeling of overexposure as well as a diminished sense of possible spatial enactment of bodily capacities. These anomalies likely stem from early neurodevelopmental disturbances in multisensory integration and sensorimotor learning. Phenomenological psychopathology further reveals how such spatial disorganization manifests as instability in self-world boundaries and a pervasive sense of altered atmosphere. Integrating neurodevelopmental, cognitive, and experiential dimensions provides a unified account of how schizotaxic vulnerability unfolds into spatial and Self-disturbances. This approach reframes embodiment and spatiality as developmental interfaces between neural processes and subjective transformation in schizophrenia.

## Introduction

Schizophrenia and its spectrum disorders profoundly reshape the structure of subjective experience^1^, particularly in how individuals perceive themselves, their bodies, their immediate environment and intersubjective atmospheres, reflecting a broader transformation of consciousness and Selfhood^2–9^. According to phenomenology, vulnerability to schizophrenia is primarily manifested in a disruption of the pre-reflective structures of embodiment and spatiality that ordinarily anchor the Self in relation to the world^1^. As such, individuals with vulnerability to schizophrenia often struggle with maintaining coherent body-centric spatial relations, leading to various alterations of the *espace vécu* (lived space) and the Self’s position within it^3^.

In agreement with such phenomenological alterations of the felt lived space around the subject (see Table 1 for a synopsis), perturbations at the spatial level are detected as body-centric metric distortions of the personal space (PS) and of the peripersonal space (PPS), i.e., key spatial boundaries that regulate interpersonal proximity and the body-environment interface^10^. PS, rooted in psycho-sociology, represents a protective interpersonal comfort-zone, while PPS, a product of neurocognitive processing, denotes the action-perception space immediately surrounding the body, demarcating a dynamic spatial boundary where sensorimotor capacities are enacted, i.e., an area in which the body’s “I-can” potentials unfold (See Glossary in Box 1).

**Table 1:** Natural experience of lived space and related, prototypical schizophrenia spectrum modifications.

Phenomenological domain	Natural experience	Experiential transformation in Schizophrenia spectrum conditions
Disorders of Spatial Coherence	Space feels seamlessly integrated; body, perception, and environment form a coherent whole. Surrounding objects fit together meaningfully.	Coherence breaks down; space becomes fragmented or void-like. Objects appear disjointed, requiring deliberate effort to piece together.
Overproximity	There is a comfortable balance between self and world, allowing for freedom, ease, and natural interpersonal spacing.	Spatial boundaries collapse, causing oppressive closeness. The environment feels overly involved and suffocating.
Self-Centrality	Perception is centered on the self but flexible, allowing shifts in perspective. The self is a reference point.	Perception becomes rigidly self-referential. The individual feels trapped at the center of space, unable to shift perspective.
Deformation of Surrounding Space	Space appears stable and relational, with distances, shapes, and proportions integrating into a coherent visual field.	Distances, proportions, and shapes are distorted. The environment can feel lifeless, static, or unnaturally vivid.
Intersubjective Stream / Intercorporeity	Shared spatiality fosters relational attunement, enabling intuitive synchronization with others and a sense of a common world.	This shared attunement breaks down. The individual feels isolated, unable to participate in a shared spatial world, leading to interpersonal alienation.
Atmosphere	Space has an affective quality that resonates with mood, offering a sense of belonging or neutrality.	Atmosphere becomes unsettling or oppressive. The environment feels hostile, ominous, or saturated with dread.
Altered Spatial Presence and Perceptual Qualities	Objects appear three-dimensional, detailed, and dynamic, revealing their nature through interaction.	Objects and spaces seem flattened, artificial, or devoid of vitality. Surfaces appear featureless, and the environment feels barren.

Box 1 Glossary **Key Concepts**

- **Lived Spatiality:** The subjective, pre-reflective experience of space as it is inhabited and navigated by the embodied self.
- **Hyper-centrality:** A phenomenological term describing a heightened self-referentiality and defensive withdrawal, often associated with expanded personal space.
- **Motoric Agency:** The pre-reflective capacity to act within the immediate spatial environment, typically instantiated within peripersonal space.
- **Embodied Self:** The minimal, non-reflective sense of self grounded in sensorimotor interaction with the environment.
- **Personal Space (PS):** A socio-emotional buffer zone around the body that regulates interpersonal proximity; linked to limbic-affective processing.
- **Peripersonal Space (PPS):** The space immediately surrounding the body where multisensory information is integrated to guide action; supported by parietal-premotor circuits.
- **PSALS:** Phenomenologically Salient Anomalies of Lived Space; a structured, non-diagnostic observational checklist summarizing key experiential features of spatial disruption in SSD.

### Neural correlates

Although both representing body-centric spatial metrics, PS and PPS are measured with different paradigms (i.e., PS via interpersonal distance tasks (finding the latest separating distance from another person feeling comfortable with), and PPS through multisensory integration tasks such as audio-tactile interference or visuo-tactile reaction paradigms) and have partially distinct putative neural correlates^11–17^. PPS is primarily encoded by a fronto-parietal network involving the premotor cortex and parietal areas (including the intraparietal sulcus and adjacent regions), where visuo-tactile and auditory signals are integrated in body-centered coordinates to guide reaching, grasping, defensive movements, and interactions with nearby objects and agents. These multisensory neurons form an adaptable, body-part-centered representation that can shift with limb position and social context. By contrast, PS regulation recruits overlapping but distinct circuits: fMRI studies show that frontoparietal regions (e.g., dorsal intraparietal sulcus and premotor cortex) respond to approaching social stimuli, and subcortical defensive systems such as the periaqueductal gray couple with premotor areas in relation to preferred interpersonal distance, reflecting affective and threat-sensitive components of social space regulation. Thus, although PPS and personal space both engage parietal-frontal networks, PPS is more tightly linked to multisensory sensorimotor integration for action, whereas personal space additionally involves social-affective and defensive processing.

### Empirical measurement

Measurable distortions of PS and PPS have been consistently observed across experimental paradigms using behavioral, neurocognitive, and psychophysiological methods, underscoring their reliability. Meta-analytical evidence reveals a consistent pattern in individuals with schizophrenia, that often exhibit an enlarged PS boundary, alongside a contracted PPS^18^. These distortions can be understood as indirect markers of a deeper reorganization of spatial experience, wherein the individual’s lived spatial field is unstable and loses it seamless natural coherence. Taking the surrounding environment as the point of reference, the enlarged PS may be interpreted as reflecting an excessive feeling of overexposure to the presence in it of other people, whereas the contracted PPS as reflecting a diminished sense of possible spatial enactment of bodily capacities, although flexibly extendable with tool use^19^. Together, these spatial anomalies reveal the contours of an underlying phenomenological disconnection between self, body, and environment, whereby the cohesiveness of one’s spatial existence erodes, leaving the individual feeling either intruded upon or detached from the immediate environment.

### A neurodevelopmental staged account

Anomalies in PS and PPS detected in patients with schizophrenia may have neurodevelopmental and neurocognitive origins. Schizophrenia is associated with impairments in multisensory and sensorimotor integration^20,21^, that are crucial processes for the ontogenesis and maintenance of PS and PPS^21,22^. These disruptions likely emerge early in development, reflecting an ontogenetic vulnerability that influences from birth the integration of bodily experience and environmental awareness^23,24^. As spatial embodiment emerges and is progressively shaped through sensorimotor learning and early caregiver interactions, early and iterated ruptures in intersubjective experiences may further hamper the ontogenesis of spatial processing^2,25–28^.

In summary, while PS and PPS are primarily non-phenomenological constructs, their distortions in schizophrenia may serve as measurable correlates of implicit and preverbal phenomenological transformations of the lived atmosphere in which the individual is immersed. These body-centric spatial anomalies signify a broader reorganization of the Self-world interface, where the embodied feeling of safety and the felt possibility of intentional action are compromised. Understanding the complex interplay between neural mechanisms, developmental trajectories, and subjective experience may provide a richer framework for exploring the prolonged pathogenetic pathways associated to the transition from schizophrenia spectrum vulnerability (e.g., schizotaxia) to more pronounced and diagnostically characterized conditions with frank psychotic symptoms. In this perspective, future studies could aim to discriminate primary alterations in body-centered metrics due to an altered ontogenetic process constrained by a fragile sensorimotor integration, and secondary alterations induced by emerging psychotic symptoms, considering a possible bidirectional influence between altered lived space and psychotic symptoms. For instance, in individuals with primary distortions of lived space, emerging psychotic symptoms such as hallucinations or paranoid delusions may amplify pre-existing PS distortions. Conversely, persecutory content might secondarily induce PS enlargement, mimicking a primary anomaly. This is preliminary confirmed by few studies assessing the relationship between PS and psychotic symptoms^29–32^.

Overall, spatial anomalies observed in schizophrenia fit into a broader matrix of neurodevelopmental and neurocognitive constraints. These constraints shape the ontogenetic trajectory of person-centered spatial cognition, ultimately influencing the pre-reflective embodied processes through which the Self, body, and world are integrated. Recognizing that PS and PPS, despite their non-phenomenological lineage, are connected to subjective spatial transformations provides a more comprehensive view of how neurodevelopmental vulnerabilities manifest in altered lived space. In the next paragraph the key concepts of such approach to the altered PS-PPS pattern in patients with schizophrenia are summarized.

### Ontogenesis and the developmental arc of spatial embodiment

To fully understand how these anomalies in felt space emerge, an ontogenetic lens is essential^27,33^. The embodied Self, ordinarily emerging through iterative sensorimotor interactions along development, provides the pre-reflective bedrock for integrating Self, body, and space: i.e., the pre-reflective (Basic or Minimal)^1^ Self becomes progressively embodied through the repetitive and consistent engagement in sensorimotor actions within the surrounding environment, coupled with contingent proprioceptive signals. Neurodevelopmental constraints linked to schizotaxic vulnerability^24,25^ likely influence the progressive embodiment of the Self and its dynamic interaction with the surrounding environment from birth. Altered neurodevelopment impacts the processing of sensory-motor stimuli - both internal and external—arising from sensorimotor exploration of the world. Ontogenetically, this may result in a partially disembodied Self and an altered *espace vécu*. From this perspective, a poorly embodied Self inherently affects the surrounding *espace vécu*, as Self and space are interconnected aspects of the same developmental process, emerging from a shared ontogenetic trajectory.

Overall, such early developmental disruptions lay the groundwork for later PS and PPS anomalies. The enlarged PS boundary could be traced back to early disturbances in sensorimotor integration, moderated by intersubjective attunement provided by early parenting, fostering a defensive stance toward others. Similarly, the reduced PPS reflects a contraction of the immediate action space, likely arising from compromised sensorimotor integration that diminishes the lived sense of “I-can.”

These disruptions cascade into the anomalous spatial experience characteristic of schizophrenia’s mature clinical presentation. While compelling, this developmental account remains a theoretical model that requires empirical validation through longitudinal and experimental approaches.

### The subjective experience of altered space: salient phenomenological features

Phenomenological psychopathology illuminates how these measurable distortions translate into psycho-existential and behavioral changes (See Table 1 for their phenomenological articulation).

As the severity progresses along the schizophrenia spectrum, the boundaries between Self, body, and world increasingly blur, leading to alterations in the subjective experience of space. These changes extend across various phenomenological domains, manifesting differently based on developmental stages and phenotypic severity within the schizophrenia spectrum disorders (SSD). Phenomenological alterations of the lived space are articulated and systematized in the Table 2 [Phenomenologically Salient Anomalies of Lived Space (PSALS)]. PSALS is a structured observational framework developed through a systematic synthesis of phenomenological literature and longitudinal clinical experience. Similar in spirit to early formulations of EASE^5^, SPI-A/CY^34^, or BSABS^35^, it maps lived spatial anomalies across developmental stages and dimensions of severity. While not a psychometric scale, its structured linguistic architecture reflects internal coherence and domain specificity. Recent developments in semantic psychometrics^36–38^ further suggest that such frameworks embed quantifiable properties even prior to formal validation, supporting their translational use in clinical and research settings.

**Table 2:** Phenomenologically Salient Anomalies of Lived Space (PSALS) and putative Schizophrenia spectrum manifestations across developmental phases.

Phenomenological level		Developmental unfolding			
Domains	Subcomponents (selective examples)	Childhood	Adolescence	Youth	Adulthood
Disorders of Spatial Coherence	Fragmentation of space. Loss of Gestalt perception. Spatial disintegration. Emptiness or disconnection	Subtle sensorimotor disruptions (clumsiness, fragmented spatial awareness). Poor object constancy (difficulty maintaining spatial unity)	Growing difficulty with spatial salience (e.g., impaired focus in cluttered spaces). Episodic fragmentation of spatial perception	Persistent disjointed spatial perception; spaces require effort to reassemble. Increasingly disorganized lived spatial field.	Chronic spatial disintegration, creating pervasive disorientation and perceptual confusion. Emergence of spatial void-like experiences.
Overproximity	Surproximité. Invasion of surrounding space. Hyper-intimacy	Hypersensitivity to crowded or cluttered environments. Early sense of spatial intrusion.	Overwhelming sense of proximity in crowded or social settings. Mild spatial boundary violations	Persistent sense of spatial intrusion, avoidance of certain spaces. Discomforting hyper-awareness of closeness or overcrowding.	Chronic collapse of spatial boundaries; pervasive spatial invasion dominates daily experiences. Severe isolation from environment.
Self-Centrality	Self as focal point. Loss of positional flexibility. Anastrophe (spatial inversion).	Early preoccupation with one’s position in space. Hyper-awareness of self as spatial center.	Episodic self-referential spatiality; feeling visibleor scrutinized.	Persistent inability to decenter oneself; increasing dominance of anastrophe (self-focused spatial inversion).	Entrenched hyper-centrality; self-centered spatiality dominates experience, isolating the individual from relational spaces.
Deformation of Surrounding Space	Altered distances or proportions. Metamorphopsia (distorted shapes). Frozen spatiality. Itemization (unnatural vividness).	Mild distortions in size, distance, or shape (e.g., objects appear briefly closer/farther).	Warped spatial proportions occur intermittently. Notable disorientation in complex settings.	Persistent deformations (e.g., objects feel disproportionate or unnaturally vivid). Episodes of frozen spatiality.	Severe and chronic spatial distortions; pervasive sense of an uninhabitable environment or dehumanized space.
Intersubjective Stream/Intercorporeity	Breakdown of shared spatiality. Alienation. Intercorporeity disruption. Loss of shared perspective.	Difficulty with joint attention(e.g., tracking gestures, engaging in shared tasks).	Emerging relational alienation; social spaces feel strained or unnatural.	Persistent alienation in shared spaces; growing disconnection from others’ spatial presence.	Total collapse of intercorporeity, creating profound relational isolation. Loss of ability to navigate social or shared spaces.
Atmosphere	Strangeness or unfamiliarity. Oppressive or threatening qualities. Hyper-salience of atmosphere.	Early fear or unease in ambiguous environments (e.g., dark or large rooms).	Spaces acquire oppressive emotional tones (e.g., foreboding or charged atmospheres).	Persistent atmospheric distortion; environments feel hostile, charged, or foreboding.	Chronic oppressive atmosphere, creating intolerable alienation or existential dread in everyday settings.
Altered Spatial Presence and perceptual qualities	Bidimensional or artifactual perception. Perception of vastness. Abnormal smoothness. Anonymity of faces or objects.	Early sense of distant or flattened world; fleeting sense of vastness or lack of detail in objects.	Objects and spaces appear flattened, lifeless, or overly smooth. Faces lack individuality (anonymity).	Persistent cinematic or artifactual perception (e.g., space feels like a movie set). Unnatural smoothness or uniformity dominates perception.	Severe bidimensionality or sense of artificiality; total detachment from a “natural” or “human” world.

Briefly, in chronic SSD conditions, profound alterations in spatial experience disrupt the natural relationship between Self, body, and environment (*Disorders of Spatial Coherence*); boundaries weaken facilitating a sense of intrusive nearness (*Overproximity*); Self-referentiality becomes preeminent (*Self-Centrality*^39^); the perceived stability of shapes and distances is distorted (*Deformation of Surrounding Space*) and the individual feels disconnected or alienated from shared spatiality (*Intersubjective Stream/Intercorporeity)*; the environment becomes imbued with uncanny and somehow menacing qualities (*Atmosphere*) and the surroundings are deprived their natural dynamic vitality (Altered Spatial Presence and Perceptual Qualities). (See Supplementary Table S1 for a structured assessment checklist aligned with current tools for prodromal/at risk mental state assessment).

These shifts in lived experience often co-occur with clinical symptoms, ranging from proactive and protective social withdrawal and more or less eccentric forms of social aversion to paranoid apprehensions, hallucinatory intrusions, and an overarching sense of a threatening atmosphere. In this context, while PS and PPS remain non-phenomenological indices, their alterations mark a phenomenological reality in which the sense of space is no longer a stable background but a source of disruption and insecurity. Table S1 provides a structured, scalable format for translating phenomenological anomalies into clinically relevant observations, supporting their integration into existing early intervention settings. While not a diagnostic tool, this framework could offer a structured way to explore how subjective spatial disturbances may present in clinical settings

### Toward an integrative framework: from neurobiology to subjective experience

An integrative framework for understanding spatial alterations in SSD presupposes multiple explanatory levels:

- *Neurobiological Level*: e.g., putative schizotaxic, neurodevelopmental vulnerability impinges on the integrity of sensory-motor circuits.
- *Neurocognitive and psychological Levels*: e.g., constrained sensory-motor processing hampers the ontogenesis of space processing and its relation with embodiment, modulated by parenting quality in supporting sensorimotor exploration and intersubjective attunement. Body-centric metrics deviations become detectable, with an expansion of PS and a contraction of PPS.
- *Subjective-Phenomenological Level*: e.g., the altered lived space reflects a fracturing of the pre-reflective unity of Self, body, and world.
- *Clinical Level*: e.g., the overall transformed experiential landscape, including anomalous spatial experiences, may consolidates clinical major symptoms such as negative and positive ones.

Across these layers, PS and PPS provide measurable metrics of spatial disruption that could be mapped along developmental phases in parallel with the progressive changes in the subjective, phenomenological dimension of experience^40^. Future research could test the proposed framework through multimodal designs combining PS/PPS paradigms with phenomenological interviews and developmental indicators. For example, longitudinal studies could examine whether early sensorimotor or intersubjective anomalies predict PS/PPS distortions and correlate with the emergence of self-disturbances or psychotic symptoms. Experimental manipulations of interpersonal space, coupled with clinical and subjective assessments, may also help operationalize key phenomenological dimensions.

Selective dialogue with adjacent frameworks, such as the spatiotemporal psychopathology model of Northoff and colleagues^41–44^, may offer reciprocal enrichment. Northoff’s approach conceptualizes psychopathology as emerging from disruptions in the brain’s intrinsic spatiotemporal dynamics - alterations in temporal continuity, spatial nestedness, and the brain–self-world alignment. Within that model, disturbances in lived time and space are not derivative but reflect global dysfunctions in neural-temporal organization. Our developmental-phenomenological model complements this perspective by independently specifying how such disruptions may become instantiated at the level of embodied spatial interfaces. PS and PPS may serve as intermediate-level spatial constructs linking intrinsic neural dynamics to lived experience, grounded in early ontogenesis and action-based embodiment. PPS, with its role in multisensory integration and action readiness, resonates with notions of temporal continuity and anticipatory structure. A contracted PPS may reflect impaired temporal binding and diminished affordances. Conversely, an enlarged PS may correspond to altered spatial nestedness and reduced intersubjective embeddedness, contributing to exaggerated self-centrality^39^ and defensive spatial postures.

Importantly, PS–PPS distortions provide operational and developmentally explorable handles for Northoff’s largely conceptual model^41–44^. While spatiotemporal psychopathology emphasizes global alterations in temporal flow and spatial organization, PS and PPS offer experimentally accessible indices of how such disruptions manifest in concrete, body-centered interactions with others and the environment. In this sense, PS-PPS distortions may be viewed as localized expressions of a more pervasive spatiotemporal disintegration making these otherwise abstract dimensions of psychopathology clinically traceable, while remaining anchored in phenomenological psychopathology and developmental neuroscience.

### Clinical implications

The proposed model may have several clinically relevant implications, particularly for early detection, phenotypic stratification, and intervention across the SSD.

The model suggests that spatial anomalies are not epiphenomenal but structurally involved in symptom formation. An enlarged personal space may predispose to social withdrawal, hypervigilance, and paranoid ideation, while a contracted peripersonal space may underlie diminished agency, motor passivity, and negative symptoms. Clinically, this implies that social avoidance, amotivation, and interpersonal mistrust may partly reflect altered embodied spatial regulation rather than purely cognitive or affective deficits. This reframing supports a further shift away from symptom-centric explanations toward disturbances of the embodied Self-world interface at the basis of symptom formation^45^.

Moreover, the PS-PPS framework opens avenues for novel, embodiment-oriented interventions. Sensorimotor training, action-based therapies, and body-focused psychotherapeutic approaches may help recalibrate distorted spatial boundaries by restoring multisensory integration and the lived sense of “I-can.” Importantly, such interventions could be deployed early, before spatial distortions consolidate into rigid experiential and behavioral patterns. From this perspective, PS and PPS metrics could serve not only as assessment tools but also as process-sensitive outcome measures for embodied and developmental interventions.

Finally, the developmental account highlights the importance of timing and staging. Spatial distortions likely evolve non-linearly across the lifespan, interacting with social context, stress, and symptom emergence. Longitudinal monitoring of PS-PPS trajectories may help disentangle primary, developmentally rooted alterations from secondary changes driven by psychotic experiences, medication, or chronicity - an issue of direct relevance for personalized treatment planning.

## Conclusions

Phenomenologists like Minkowski and Jaspers long ago emphasized that spatial disorganization is central to schizophrenia’s core psychopathology. Contemporary research, bridging phenomenology with psycho-sociological and neuroscientific approaches, corroborates that this spatial disruption is neither random nor peripheral. Instead, it reflects fundamental neurocognitive and neurodevelopmental vulnerabilities that impinge directly upon subjective experience. By integrating phenomenological insights with non-phenomenological metrics like PS and PPS, we gain a comprehensive framework that acknowledges both the measurable and the lived. This synergy may clarify how neurodevelopmental constraints, embodied processes, and subjective transformations coalesce to produce the characteristic spatial dysfunctions of schizophrenia.

The systematic distortion of PS and PPS may offer important insights on the developmental unfolding of vulnerability to SSD. Indeed, although these constructs are not phenomenological in nature, their perturbations closely align with subjective transformations of spatial experience. Crucially, these transformations occur at a tacit, pre-reflective level of experience, representing an overarching qualitative reconfiguration of the very structure of experiencing, with PSALS (i.e., Phenomenologically Salient Anomalies of Lived Space) that could be also tied with age-specific changes in PS and PPS organization. At the same time, the present framework preserves a critical distinction between body-centric spatial constructs - such as PS and PPS - and the phenomenological domain of lived spatiality. PS and PPS are not lived space per se, but rather operationalized, non-phenomenological indices that acquire relevance through their ontogenetic entanglement with the embodied Self. Maintaining this stratification allows for conceptual clarity and avoids premature unification across explanatory levels, while still encouraging translational bridges between phenomenology and neuroscience. Indeed, this framework offers a novel path for integrating experimental data, developmental neuroscience, and lived experience in schizophrenia and vulnerability to spectrum disorders. By bridging the measurable (i.e., body-centric metrics such as PS and PPS) with the experiential (altered embodiment and spatiality), we propose a translational model that not only enriches theoretical understanding but also opens the door to new assessment tools and intervention strategies.

### Future directions

Future work can build on this foundation to develop clinically relevant markers of early spatial disorganization, refine phenomenologically informed interviews, and empirically test how these embodied disturbances unfold over time. Spatial metrics and their embodied, sensorimotor sense of coherence, may thus serve as a developmental interface linking brain, body, and subjectivity in the complex trajectory of schizophrenia spectrum vulnerability. Future work may explore how PS/PPS anomalies manifest across other psychiatric conditions (e.g., mood and anxiety disorders), allowing finer differential diagnostics^46^.

## Supplementary information

Table S1
