# Supplementary material for: Spatial disruptions and the embodied Self in schizophrenia: toward a developmental framework
Source: Schizophrenia (Heidelb). 2026 May 5;12(1):62. doi: 10.1038/s41537-026-00749-8 (PMC13350905; doi:10.1038/s41537-026-00749-8)
Supplement: Supplementary file 1 — Table S1 [file 41537_2026_749_MOESM1_ESM.docx]

**Table S1.** Phenomenologically salient anomalies of lived space (PSALS) checklist, integrating severity, intensity, interference, and distress as key criteria.

**Key features:**

**Severity Integration**: The 0–6 scale, aligns with contemporary CAARMS/SIPS/PSYCHS conventions, allowing for straightforward integration into diagnostic or therapeutic settings and ensuring the due granularity in rating both subtle and severe presentations of spatial anomalies.

Each phenomenological theme progresses from normal experiences to total dominance by spatial disturbances.

| **Phenomenological Levels** | | **Scores** | | | | | | |
| --- | --- | --- | --- | --- | --- | --- | --- | --- |
| **Theme** | **Subcomponents**  **(selective examples)** | **0 - Absent** | **1 - Questionable** | **2 - Mild** | **3 - Moderate** | **4 - Marked** | **5 - Severe (Not Psychotic)** | **6 - Psychotic/**  **Very Severe** |
| **Disorders of Spatial**  **Coherence** | Fragmentation of space, loss of Gestalt, spatial disintegration, emptiness/  disconnection | No spatial anomalies observed/reported. | Fleeting, vague spatial disarray; questionable symptoms. | Mild fragmentation; occasional difficulty maintaining spatial coherence. | Episodic fragmentation; requires mental effort to restore spatial unity. | Persistent fragmentation; frequent interference with spatial focus. | Chronic disintegration of spatial coherence; space feels persistently disorganized. | Complete spatial disintegration; pervasive chaos and emptiness dominate spatial perception. |
| **Overproximity** | Surproximité, invasion of surrounding space, hyper-intimacy | Normal sense of personal space. | Vague discomfort with closeness; questionable sense of intrusion. | Mild sense of proximity discomfort; limited to specific situations. | Episodic sense of spatial intrusion, creating discomfort in social or crowded spaces. | Persistent sense of overproximity; spatial invasion disrupts daily life. | Chronic collapse of spatial boundaries; environment feels unbearably close. | Total invasion of surrounding space; intolerable closeness and disorientation dominate. |
| **Self-Centrality** | Self as focal point, loss of positional flexibility, anastrophe | No self-referential spatial experiences. | Subtle fixation on one’s spatial position; fleeting sense of being central. | Mild hyper-self-referentiality; occasional difficulty shifting spatial focus. | Episodic fixation on self as spatial center; interference with perspective-taking. | Persistent dominance of self-centered spatiality; spatial focus is rigidly self-referential. | Entrenched anastrophe; self-referential spatiality dominates experience. | Total inversion of spatial relations; space is fully organized around the self. |
| **Deformation of Surrounding Space** | Altered distances/proportions, metamorphopsia, frozen spatiality, itemization | Normal perception of spatial dimensions. | Vague, fleeting distortions in distance, size, or proportion. | Mild perceptual anomalies; limited impact on spatial understanding. | Episodic distortions; objects occasionally feel warped, frozen, or overly salient. | Persistent deformation of space; significant interference with navigation or interaction. | Chronic and severe spatial warping; distances, proportions, and object relationships feel unreal. | Complete spatial deformation; space becomes uninhabitable and detached from reality. |
| **Intersubjective Stream/**  **Intercorporeity** | Breakdown of shared spatiality, alienation, intercorporeity disruption | Normal intersubjective spatial experiences. | Fleeting difficulty with joint attention or shared spatiality. | Mild disconnection from shared space; intermittent relational alienation. | Episodic breakdown in intercorporeity; difficulty sustaining relational spatiality. | Persistent relational alienation; shared spaces feel strained or unnatural. | Severe collapse of intercorporeity; profound isolation from shared spatial environments. | Total disconnection from relational spatiality; inability to engage with others in shared space. |
| **Atmosphere** | Strangeness, unfamiliarity, oppressive qualities, emotional charge | Normal emotional tone of environments. | Vague sense of unease or strangeness in certain spaces. | Mild emotional tension in spaces; limited discomfort. | Episodic oppressive or foreboding atmospheres; spaces intermittently feel hostile. | Persistent emotional distortion; spaces feel charged with unease, tension, or hostility. | Chronic oppressive atmosphere; environments are consistently alien or threatening. | Spaces become unbearably foreboding, alien, or menacing; profound emotional distress dominates. |
| **Altered Spatial Presence and perceptual qualities** | Flattened, bidimensional, or artifactual perception; perception of vastness, smoothness, anonymity | Normal perception of objects and space. | Subtle sense of spatial flatness or distance; questionable alterations. | Mild sense of bidimensionality or surface abnormality; minimal discomfort. | Episodic flattening or cinematic perception; surfaces or spaces feel subtly artificial. | Persistent dimensional or artifactual perception; significant alienation from natural textures. | Chronic sense of artificiality; world feels overly smooth, lifeless, or staged. | Total detachment from natural space; pervasive bidimensionality or artificiality dominates experience. |

**Severity Scale Anchors (derived from CAARMS/SIPS/PSYCHS conventions)**

**0 – Absent**: No anomalies observed or reported.

**1 – Questionable**: Subtle, vague, or fleeting symptoms; easily dismissed.

**2 – Mild**: Noticeable but tolerable anomalies; limited distress or interference.

**3 – Moderate**: Episodic and distressing anomalies; manageable with effort; mild functional impairment.

**4 – Marked**: Persistent symptoms that significantly interfere with perception and cause distress.

**5 – Severe (Not Psychotic)**: Chronic, severe anomalies that dominate perception but lack psychotic certainty; major functional impairment.

**6 – Psychotic/Very Severe**: Total and pervasive dominance of anomalies; psychotic-level immersion, extreme distress, and severe functional impairment.
